# Increased Kindlin-2 via SMURF1 Inhibition Attenuates Endothelial Permeability and Acute Lung Injury

**DOI:** 10.3390/ijms26051880

**Published:** 2025-02-22

**Authors:** Weiguo Chen, Yulia Epshtein, Christen Vagts, Anne E. Cress, Jeffrey R. Jacobson

**Affiliations:** 1 Department of Medicine, Division of Pulmonary, Critical Care, Sleep & Allergy, University of Illinois Chicago, Chicago, IL 60612, USA; 2 Department of Cellular and Molecular Medicine, University of Arizona, Tucson, AZ 85724, USA

**Keywords:** kindlin-2, SMURF1, endothelial cells, acute lung injury

## Abstract

Integrin β4 (ITGB4) mediates lung endothelial cell (EC) inflammation attenuated by simvastatin, an HMG CoA-reductase inhibitor. The cytoplasmic domain of ITGB4 is predicted to bind kindlin-2. Kindlin-2 expression is mediated by SMURF1, an E3 ubiquitin ligase that promotes kindlin-2 ubiquitination and degradation. We hypothesized that increased kindlin-2 expression via the inhibition of SMURF1 mediates EC inflammatory responses relevant to acute lung injury (ALI). To investigate this, human lung ECs were treated with simvastatin (5 µM, 16 h) prior to the immunoprecipitation of kindlin-2 and Western blotting for ITGB4. Next, ECs were treated with a SMURF1 inhibitor, A01, and increased kindlin-2 expression was confirmed. In assays of barrier function, kindlin-2 was silenced (siRNA) in ECs prior to thrombin and measurements of transendothelial resistance (TER) and FITC-dextran transwell flux. Repeat assessments of barrier function were performed in A01-treated ECs. Finally, mice were pretreated with A01 prior to LPS; bronchoalveolar lavage (BAL) fluid was collected, and their lungs were used for histology. Simvastatin increased ITGB4:kindlin-2 association, while A01 increased kindlin-2 expression. Thrombin-induced EC barrier disruption was both increased after kindlin-2 silencing and decreased by A01. Finally, murine ALI was significantly attenuated by A01. Our findings suggest that the augmentation of kindlin-2 may serve as a novel ALI therapeutic strategy.

## 1. Introduction

We previously identified integrin β4 (ITGB4) as a mediator of the vascular-protective properties of simvastatin, an HMG co-A reductase inhibitor, via effects on endothelial cell (EC) inflammatory responses relevant to acute lung injury (ALI) [1,2]. Evidence in support of this idea includes a robust increase in EC ITGB4 expression after simvastatin treatment that is also associated with an attenuation of ITGB4 tyrosine phosphorylation. These effects are inhibited by an anti-ITGB4 antibody that we found augments ITGB4 tyrosine phosphorylation. Furthermore, the overexpression of ITGB4 is associated with increased lung EC inflammatory responses, while mice expressing a mutant ITGB4 lacking a cytoplasmic tail are protected in a murine model of ALI [3]. However, the mechanisms by which ITBG4 meditates protection in ALI models remain poorly characterized.

The integrin beta family of proteins are transmembrane proteins, amongst which ITGB4 is characterized by a cytoplasmic tail of over 1000 amino acids that is uniquely long relative to the other intergrin β subunits. This cytoplasmic domain includes structural elements which allow ITGB4 to bind to plectin, the adapter protein that links ITGB4 with the cytoskeleton [4,5]. In addition, integrins are known components of focal adhesions, and ITGB4 is involved in the activation of focal adhesion kinase (FAK) [6], a mediator of EC barrier function. Notably, the kindlin family of proteins is recognized to regulate integrins and the assembly of focal adhesions [7,8]. Furthermore, amino acid sequences within the ITGB4 cytoplasmic domain are predicted to bind kindlin-2 [9,10,11]. Kindlin-2 has been identified as a mediator of EC barrier function, with decreased expression being associated with increased EC permeability [12]. However, evidence of an attenuation of EC permeability associated with increased kindlin-2 expression has not been previously reported nor has it been studied in the context of ALI. We hypothesized that the augmentation of kindlin-2 augments lung EC barrier function and is associated with an attenuation of lung vascular permeability associated with ALI.

Kindlin-2 expression levels are mediated by SMURF1 (Smad ubiquitination regulatory factor-1), an E3 ubiquitin ligase that promotes kindlin-2 ubiquitination and degradation [13]. Furthermore, SMURF1 expression is inversely correlated with integrin activation. A01 is a small molecule inhibitor of SMURF1 that inhibits E3 ligase function through the binding of the SMURF1 WW1 domain, and it is required to recognize some substrates for degradation [14]. In this study, we investigated the role of kindin-2 in EC barrier function in vitro and the effects of kindlin-2 augmentation via A01, both in vitro and in vivo, in a murine model of ALI.

## 2. Results

### 2.1. Association of Kindlin-2 and ITGB4 in Human Lung Endothelial Cells (EC) and Effects of SMURF1 Inhibition on Kindlin-2 Expression

**Figure 1:**
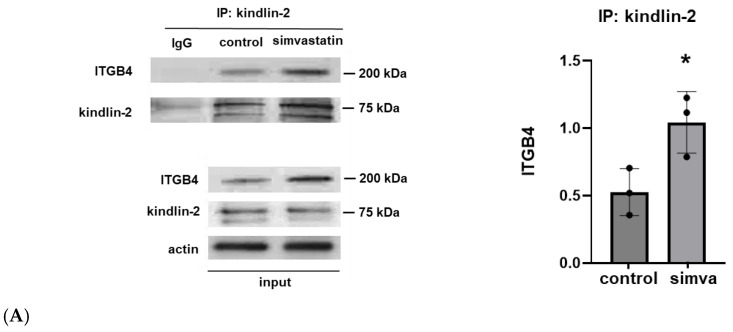

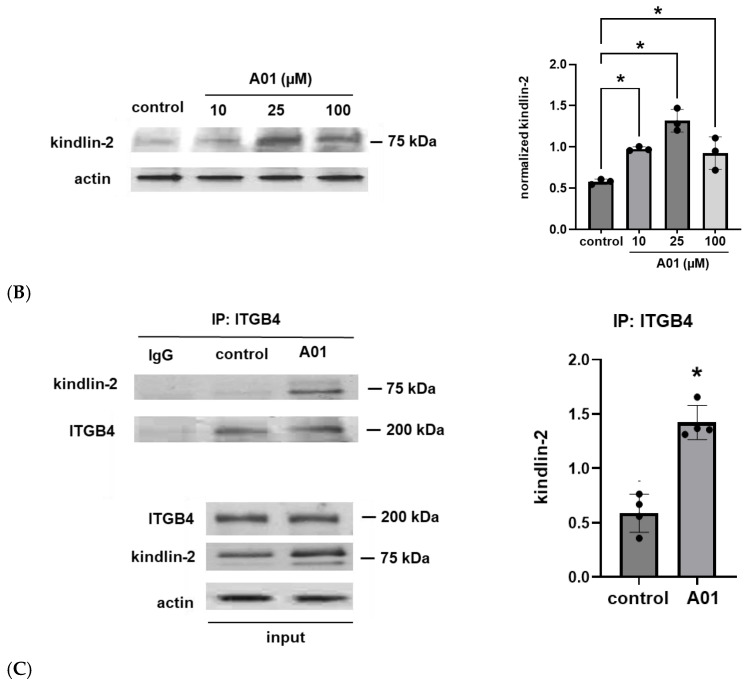
Kindlin-2 and ITGB4 co-association and increased kindlin-2 expression after SMURF1 inhibition. (**A**) Human pulmonary artery ECs were treated with simvastatin (5 µM for 16 h) prior to IP of kindlin-2 and Western blotting for ITGB4 (n = 3/condition, * *p* < 0.05). In separate experiments, ECs were treated with the SMURF1 inhibitor, A01 (0–100 µM for 3 d), followed by (**B**) Western blotting of whole cell lysates for kindlin-2 or (**C**) the immunoprecipitation of ITGB4 followed by Western blotting for kindlin-2 (n = 3–4/condition, * *p* < 0.05). Representative blots are shown, including Western blots from whole cell lysates used for loading controls.

In the initial experiments, human pulmonary artery ECs were treated with simvastatin (5 µM for 16 h) to induce ITGB4 expression as we have previously reported [1]. Cell lysates were then utilized for the immunoprecipitation of kindlin-2 with Western blotting for ITGB4. These experiments demonstrated a significant increase in EC ITGB4:kindlin-2 association induced by simvastatin. Subsequently, ECs were treated with A01, and increased kindlin-2 expression was confirmed by Western blotting with a dose-dependent response noted at lower A01 concentrations (10 and 25 mM) that was not evident at higher concentrations (100 mM). In separate experiments, IP of ITGB4 also confirmed increased ITGB4:kindlin-2 association after A01 treatment (Figure 1).

### 2.2. The Effects of Kindlin-2 Depletion on Human Lung EC Barrier Function

**Figure 2:**
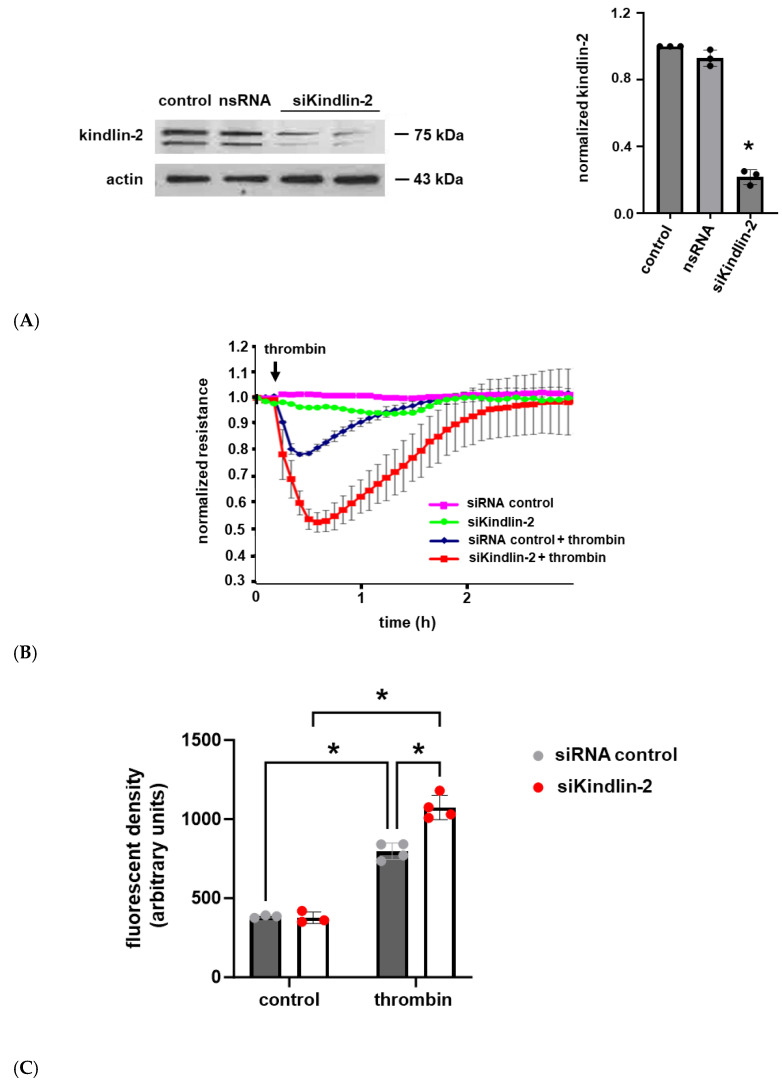
Thrombin-induced human lung EC barrier disruption is increased after kindlin-2 knockdown. (**A**) Human pulmonary artery ECs were transfected with siRNA specific for kindlin-2 (100 nM for 3 d) or control siRNA, and lysates were used for Western blotting to confirm kindlin-2 knockdown. (**B**) Control and kindlin-2-silenced ECs grown to confluence overlying gold-plated microelectrodes and transendothelial electrical resistance were measured over time after treatment with thrombin (1 U/mL). (**C**) In separate experiments, controls and kindlin-2-silenced ECs were grown in transwell inserts with FITC-labeled Dextran applied to the top surface prior to treatment with thrombin (1 U/mL for 1 h) The medium underneath the transwells was then collected and assessed for fluorescence density (n ≥ 3/condition; * *p* < 0.05).

To explore the functional role of kindlin-2 in human lung EC barrier regulation, cells were transfected with siRNA specific for kindlin-2 with silencing confirmed by Western blotting (Figure 2A). Cells were grown to confluence overlying gold-plated microelectrodes and then treated with thrombin (1 U/mL) to induce barrier disruption with EC barrier integrity and then assessed by real-time measurements of transendothelial electrical resistance (TER). These experiments confirmed a significant increase in thrombin-induced barrier disruption with a delayed recovery to baseline in kindlin-2-silenced ECs compared to controls (Figure 2B). In complementary experiments, kindlin-2-silenced ECs were grown in transwell inserts, and FITC-Dextran transwell flux was measured after thrombin stimulation (1 U/mL for 1 h). EC monolayer permeability was significantly increased in kindlin-2-silenced ECs compared to thrombin-stimulated control cells transfected with non-specific siRNA (Figure 2C). Together, these experiments are consistent with the idea that kindlin-2 promotes EC barrier integrity in response to inflammatory stimuli.

### 2.3. The Effects of Kindlin-2 Augmentation via SMURF1 Inhibition on Human Lung EC Barrier Function

**Figure 3:**
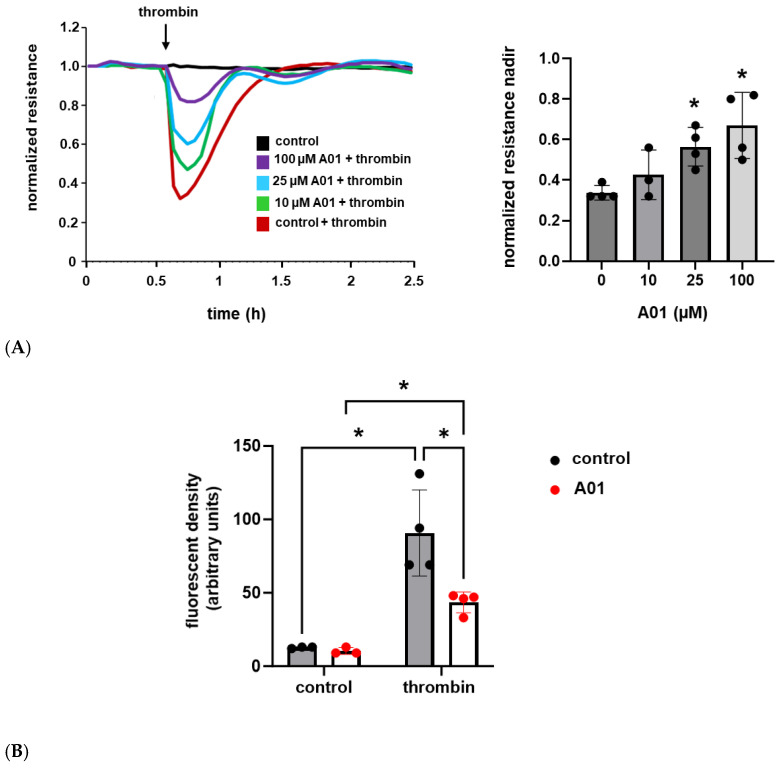
A01 attenuates thrombin-induced EC barrier disruption. (**A**) Human pulmonary artery ECs were grown to confluence overlying gold-treated microelectrodes and treated with A01 (0–100 µM for 3 d) prior to thrombin (1 U/mL) stimulation with measurements of transendothelial resistance (representative tracing shown; n = 4, * *p* < 0.05 compared to thrombin-treated controls). (**B**) Separately, ECs were grown in transwell inserts and treated with A01 (25 µM for 3 d) prior to thrombin (1 U/mL for 1 h) and measurements of FITC–Dextran monolayer flux (n ≥ 3/condition; * *p* < 0.05).

Next, we used A01 to augment kindlin-2 expression levels and utilized the same assays to assess the effects on EC barrier function. A01 treatment was associated with a dose-dependent response with a significant attenuation of thrombin-induced EC barrier dysfunction and a more rapid recovery to baseline as measured by TER, while EC monolayer FITC–Dextran transwell flux in response to thrombin was markedly decreased in A01-treated ECs compared to untreated controls (Figure 3).

### 2.4. SMURF1 Inhibition via A01 in Murine ALI

**Figure 4:**
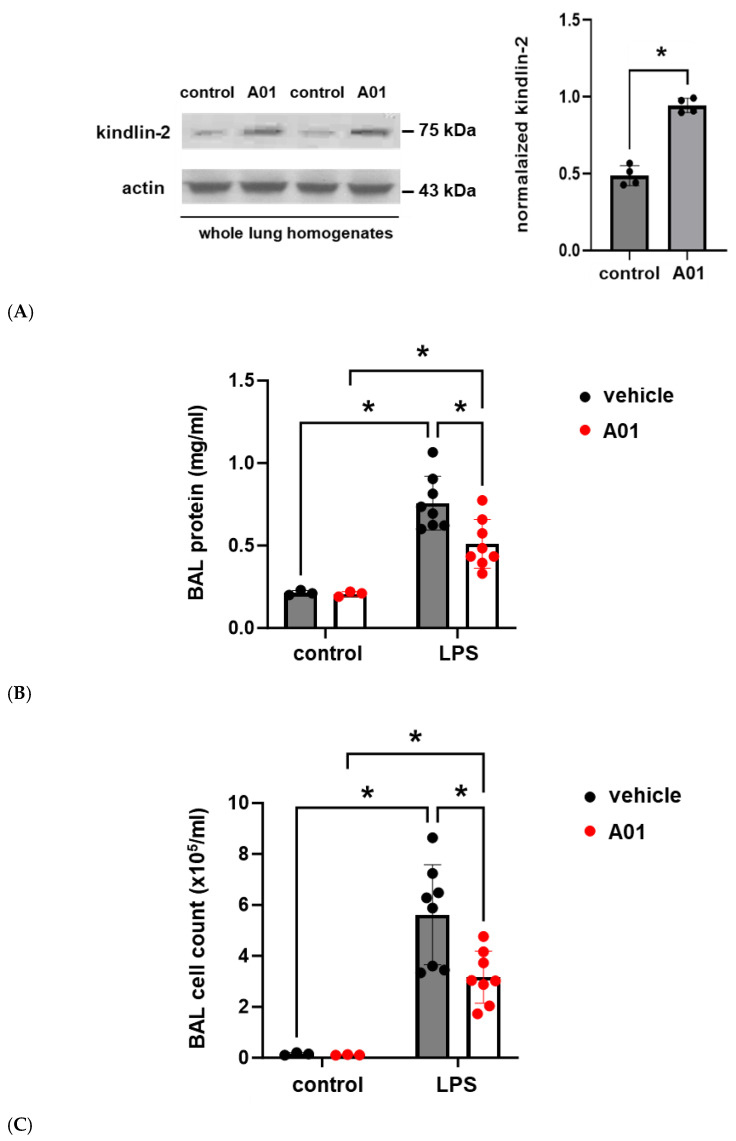
Murine ALI is attenuated by A01: BAL protein and cell counts. Mice were pretreated with A01 (0.1 mg/kg, IP) or vehicle for 48 h prior to repeat treatments under the same conditions, with LPS (1.25 mg/kg) given by intratracheal administration 1 h later to induce ALI. At 24 h after LPS, the animals were sacrificed, and BAL fluid was collected for measurements of the total protein and inflammatory cells, which are indices of lung injury. (**A**) Whole lung homogenates from control and A01-treated mice were subjected to Western blotting for kindlin-2 (representative blots shown; n = 4/condition; * *p* < 0.05). Separately, BAL fluid was used to measure (**B**) total protein and (**C**) cell counts (n ≥ 3/condition; * *p* < 0.05).

**Figure 5:**
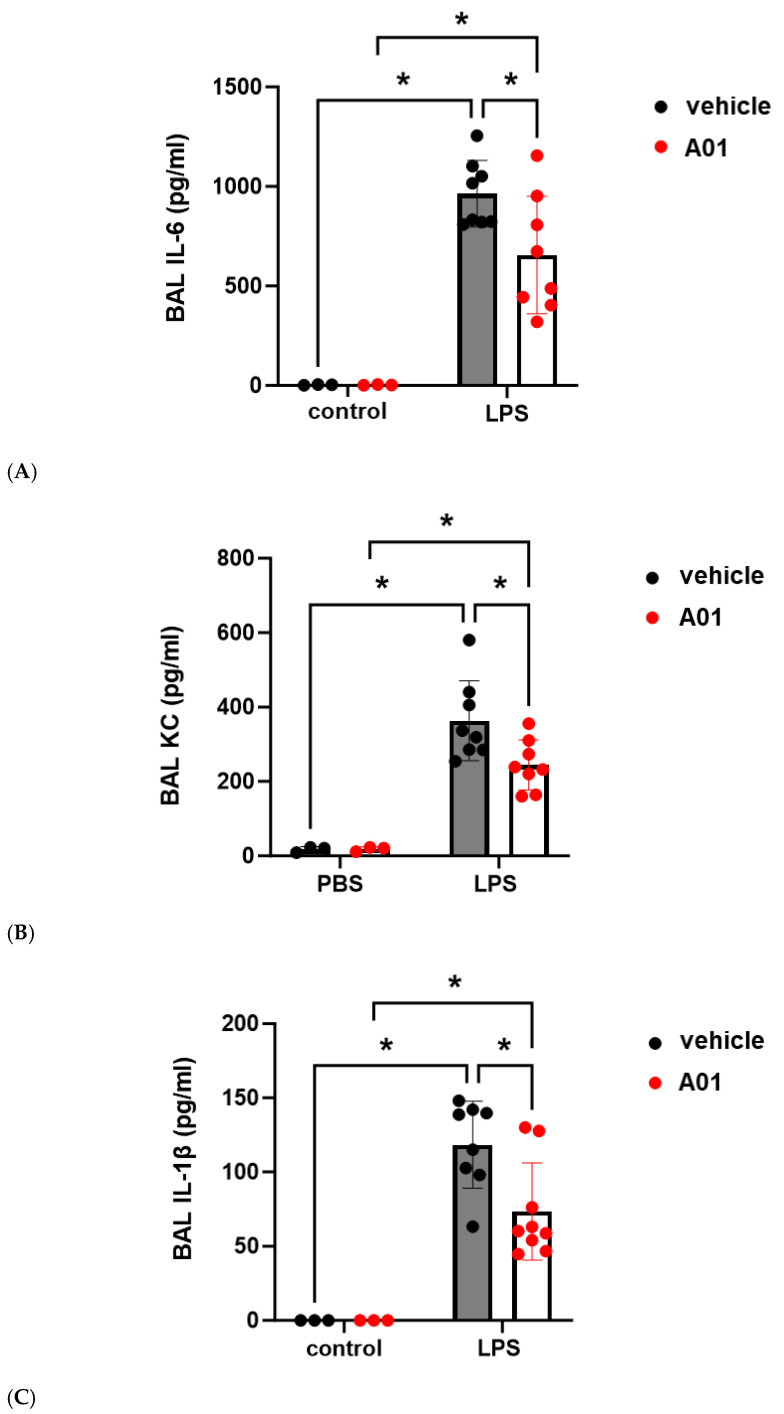
Murine ALI is attenuated by A01: BAL cytokines. Mice were pretreated with A01 (0.1 mg/kg, IP) or vehicle for 48 h prior to repeat treatments of the same for each animal, with LPS (1.25 mg/kg) given by intratracheal administration 1 h later to induce ALI. At 24 h after LPS, the animals were sacrificed, and BAL fluid was collected to measure cytokine levels, (**A**) IL-6, (**B**) KC, and (**C**) IL-1β, which are indices of inflammation (n ≥ 3/condition; * *p* < 0.05).

To explore the role of kindlin-2 in lung vascular inflammatory responses in vivo, mice were pretreated with A01 (0.1 mg/kg, IP) 48 h and again 1 h prior to the administration of intratracheal LPS (1.25 mg/kg). Western blots of whole lung homogenates confirmed increased kindlin-2 expression after A01, and bronchoalveolar lavage (BAL) fluid was collected 24 h after LPS and used to measure the protein and total cell counts (Figure 4). Notably, A01 treatment was associated with a significant reduction in LPS-induced protein and total cell counts compared to the controls. Separately, inflammatory cytokines were also measured in the BAL fluid after LPS treatment and confirmed significant reductions in several inflammatory cytokines in A01-treated animals compared to the LPS controls, including IL-6, KC, and IL-1β (Figure 5).

**Figure 6:**
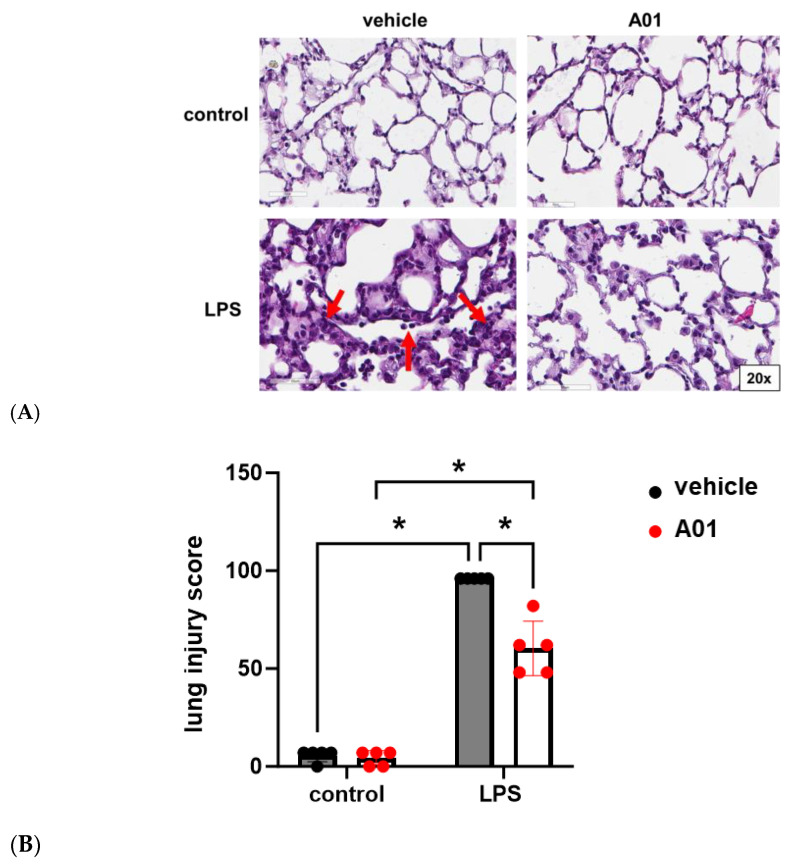
Murine ALI is attenuated by A01: lung histology. (**A**) H&E staining was performed, and representative images from the lungs of mice treated with A01 or vehicle prior to LPS administration are shown. Compared to the controls, increased interstitial edema with inflammatory cell infiltration was seen after LPS (bottom left panel, arrows). These changes were markedly reduced in animals pretreated with A01 prior to LPS (bottom left). (**B**) Lung injury scores are shown (n = 5/condition; * *p* < 0.05).

Finally, from the same animal experiments, lungs were harvested, and H&E staining was performed for a histological evaluation and lung injury scoring (Figure 6). Histology confirmed increased interstitial edema and inflammatory cells after LPS, consistent with ALI, which was markedly attenuated by A01. Consistent with these observations, A01 was associated with a significant attenuation of LPS-induced ALI, as assessed by lung injury scoring relying on the degree of neutrophil prevalence, hyaline membranes, airspace proteinaceous debris, and alveolar septal thickening.

## 3. Discussion

Strategies aimed at mitigating lung endothelial cell (EC) permeability have garnered significant interest due to the lack of effective therapeutics for acute lung injury (ALI). We have previously identified ITGB4 as one potential therapeutic target as it is a robust mediator of lung EC inflammatory responses, although the mechanisms involved have not been fully characterized. ITGB4 can bind kindlin-2, a component of EC focal adhesions that has also been reported to interact with EC adherens junctions to mediate EC barrier function [12]. Our results confirm both an association of ITGB4 with kindlin-2 in lung ECs and a correlation between kindlin-2 expression levels and effects on lung vascular permeability associated with ALI protection.

Studies designed to characterize a functional role for kindlin-2 in EC biology have previously identified effects on angiogenesis. For example, EC migration and tube formation are promoted through kindlin-2 effects on VEGF-A, and EC-specific kindlin-2 knockout embryos die in utero due to impaired angiogenesis and hemorrhage [15]. In addition, kindlin-2 ± mice demonstrate abnormal angiogenesis with immature and leaky vessel formation [16]. Separately, integrin-mediated signaling modulated by kindin-2 is known to involve ITGB1 and ITGB3. Acetylation of the ITGB1 cytoplasmic tail promotes kindlin-2 binding and differential EC gene expression associated with cell adhesion, proliferation, and barrier function [17]. Kindlin-2 also serves as a co-activator with talin, a cytoskeletal actin-binding protein, to target ITGB3 [18]. Notably, kindlin-2 mediates reciprocal ITGB1 and ITGB3 antagonism with effects on EC adhesion and contractility through activation of the small GTPase, RhoA [19].

An association of kindlin-2 with ITGB4 in ECs and its functional significance has not previously been characterized. However, interactions between β integrin subunits and both kindlin-2 and talin are known to induce conformational changes in integrin cytoplasmic tails that promote protein activation [20]. Furthermore, we previously identified a number of downstream effects of ITGB4 activation associated with EC barrier augmentation, including effects on mitogen-activated protein kinase (MAPK) signaling mediated by the protein tyrosine phosphatase SHP-2 [2], the activation of Rac GTPase, as well as complex formation with c-Met, a receptor tyrosine kinase, and sphingosine 1-phosphate receptor 1, which serves to augment EC barrier integrity [21]. Moreover, kindlin-2 binds endothelial adherens junctional components directly, including β catenin and actin [12], suggesting that multiple events likely contributed to the effects we observed.

To augment kindlin-2, we utilized A01, a small molecule inhibitor of SMURF1. SMURF1 is an E3 ligase that targets kindlin-2 for degradation via ubiquitination. The use of A01 in our studies to attenuate murine ALI supports the translational potential of our findings. In this regard, it is notable that there were no observable adverse effects or toxicity associated with A01 in our murine studies. Furthermore, SMURF1^−/−^ mice have no discernible phenotype and have normal vitality and fertility [22]. Moreover, the idea of SMURF1 inhibition in patients has gained interest as a potential therapeutic strategy in other clinical contexts including pulmonary hypertension [23,24] and macular degeneration [25]. However, we recognize that there are important qualifiers associated with this idea that must be studied before its translational potential can be realized, including the potential off-target effects of A01 as well as effects on other target proteins mediated by SMURF1. This idea is suggested by our observation of dose-dependent EC barrier protection affected by A01 although the effects of A01 on increased kindlin-2 expression were not as significant at the higher concentrations studied. One possible explanation for this finding is that A01 also affects other proteins involved in EC barrier regulation. Although we have conducted studies that found no effect of A01 on the expression of VE-cadherin, a core component of EC adherens junctions, additional experiments are underway to investigate the potential effects on other proteins.

A proline-rich (PY) motif on substrate proteins enables SMURF1 targeting [26]. Consistent with this, a kindlin-2 PY motif mutant was found to be resistant to SMURF1-mediated degradation [13]. SMURF1 contains an N terminal C2 domain, followed by two WW domains, and a HECT domain at the C terminus. Interestingly, A01 specifically binds the WW1 domain, although PY-motif ligand binding is only found in the second of the two SMURF1 WW domains, WW2. However, this is also true of the third of three SMURF2 WW domains, although both WW2 and WW3 are requisites for SMURF2 protein targeting [26]. We speculate that, like SMURF2, both WW domains are requisites for effective SMURF1 protein targeting, which would account for the increased EC kindlin-2 expression levels we observed with the administration of A01.

Taken together, our findings in this study and our previously published results support the idea that increased kindlin-2 expression attenuates lung EC inflammatory responses through effects mediated by ITGB4. It should be noted that ITGB1 and ITGB3, both kindlin-2 targets, have also been implicated as mediators of EC permeability in ALI. However, ITGB1 exacerbates inflammation and vascular permeability in ALI models, while antibodies against ITGB1 are protective in this context [27,28]. Separately, ITGB3 is known to promote NF-κB-mediated inflammatory signaling, and ITGB3 knockdown attenuates lung vascular permeability in ALI models [29]. In addition, increased ITGB3 expression and activation is associated with increased LPS-induced lung fibrosis in a murine model [30]. These reports suggest that the protective effects of kindlin-2 augmentation that we observed in our experiments are not mediated through the increased activation of ITGB1 nor ITGB3. Nonetheless, efforts to establish a definitive mechanistic link between ITGB4 and kindlin-2 in our studies are a focus of ongoing research.

Kindlin-2 has been identified as a mediator of a number of disease processes, making it an attractive therapeutic target in a variety of different clinical contexts, including osteoporosis [31,32], diabetes [33], and cancer [34]. Our results suggest that ALI may also be amenable to treatment or prevention through targeting kindlin-2. The inhibition of SMURF1 to augment kindlin-2 expression represents one such strategy. The use of A01 as a novel therapeutic strategy for ALI now warrants further study, as do efforts to define the mechanisms underlying the regulation of EC kindlin-2, which may lead to more precise effective approaches to target kindlin-2 in ALI.

## 4. Materials and Methods

### 4.1. Antibodies and Reagents

Antibodies against ITGB4 (Santa Cruz Biotechnology, Santa Cruz, CA, USA), kindlin-2 (Cell Signaling, Danvers, MA, USA) and β-actin (Sigma, St. Louis, MO, USA), were purchased from the indicated vendors. The SMURF1 inhibitor A01 was purchased from Millipore Sigma (Burlington, MA, USA) and prepared in DMSO. Kindlin-2 silencing RNA (siRNA) was purchased from GE Dharmacon (Lafayette, CO, USA). All other reagents were purchased from Sigma unless otherwise indicated.

### 4.2. Endothelial Cell Culture

Human pulmonary artery endothelial cells (ECs) were cultured in essential growth medium (EGM-2) containing 10% fetal bovine serum (Clonetics, Walkersville, MD, USA). Cells were grown to confluence in an incubator at 37 °C with 5% CO_2_ and 95% humidity.

### 4.3. Endothelial Cell (EC) siRNA Transfection

ECs were transfected with siRNA using transfection reagent siPORT Amine (Ambion, Austin, TX, USA) in serum-free conditions according to the manufacturer’s protocol. Media was changed to EGM-2 containing 2% fetal bovine serum after 24 h of transfection, and protein silencing was evaluated after 72 h.

### 4.4. Immunoblotting and Immunoprecipitation

Western blotting was performed using standard protocols, and bands densities were measured using ImageJ (v 1.54f, National Institutes of Health, http://imagej.nih.gov/ij/, accessed on 9 September 2024)). For immunoprecipitation, proteins were extracted using standard protocols, and immunoprecipitation was performed followed by Western blotting according to standard protocols.

### 4.5. Transendothelial Electrical Resistance (TER) Measurement

ECs were cultured in polycarbonate wells containing gold microelectrodes to measure TER using an electric cell–substrate impedance system (Applied Biophysics, Troy, NY, USA) as previously described [35]. TER values were pooled at discrete time points and plotted against time as the mean ± SEM.

### 4.6. Transwell Permeability Assay

A commercially available kit (Millipore, Billerica, CA, USA) was used to measure EC monolayer transwell permeability as we have previously described [36]. Thrombin (1 U/mL) and FITC–dextran (2000 kD, 100 µL) were added to ECs grown to confluence in transwell chambers and incubated for 1 h. Media (100 μL) were collected, and fluorescent density was analyzed on a Titertek Fluoroskan II Microplate Fluorometer (Diversified Equipment, Lorton, VA, USA) at excitation and emission wavelengths of 485 and 530 nm, respectively.

### 4.7. Murine Acute Lung Injury (ALI) Model

All experiments and animal care procedures were approved by the UIC Animal Care and Use Committee (ACC protocol #24-129, approval date 10 July 2024) and in accordance with the Guide for the Care and Use of Laboratory Animals, Eighth Edition, published by the Institute for Laboratory Animal Research. Male 8- to 12-week-old C57BL/6 mice (Jackson Laboratory, Bar Harbor, ME, USA) were treated with A01 (0.1 mg/kg, IP) or vehicle for 48 h prior to repeat treatments of the same for each animal, with lipopolysaccharide (LPS, 1.25 mg/kg, IT) given 1 h later. At 24 h, bronchoalveolar lavage (BAL) fluid was collected and assessed for total protein as well as total cell counts as previously described [37]. Separately, lungs were harvested for histologic evaluation, and lung injury scoring was performed by a blinded evaluator in accordance with the American Thoracic Society guidelines [38].

### 4.8. Statistics

All results are expressed as mean ± SE. Statistical analysis was conducted using unpaired Student’s *t*-test to compare the means of the data. Significant differences among multiple groups were confirmed by a one-way ANOVA followed by Tukey’s multiple comparisons test. *p*-values less than 0.05 were considered statistically significant.

## Data Availability

The data that support the findings of this study are available from the corresponding author upon reasonable request.

## Abbreviations

The following abbreviations are used in this manuscript:

ALI	acute lung injury
BAL	bronchoalveolar lavage
EC	endothelial cells
FAK	focal adhesion kinase
FAK	focal adhesion kinase
ITGB1	integrin β1
ITGB3	integrin β3
ITGB4	integrin β4
LPS	lipopolysaccharide
siRNA	silencing RNA
SMURF1	smad ubiquitination regulatory factor-1
TER	transendothelial electrical resistance
